# Practical and Analytical Chemistry

**Published:** 1885-10

**Authors:** 


*Practical and Analytical Chemistry.*—Being a Complete Course in Chemical Analysis. By Henry Trimble, Ph. G., Professor of Analytical Chemistry in the Philadelphia College of Pharmacy. Illustrated. Publishers: P. Blackiston, Son & Co., Philadelphia. 1885. Price, $1.50.

The object of this excellent work is to place before the student of pharmacy and medicine a compact course of analytical chemistry.

The distinguished author believing that the study of Qualitative Analysis should be preceded by some practical experience such as relates to the preparation of the important gases and a few of the salts, devotes Part First to the consideration of Hydrogen, Chlorine, Hydrochloric Acid, Oxygen, Nitrogen, Ammonia, Nitric Acid, Carbon Dioxide and the preparation of such salts as Potassium Chloride, Potassium and Sodium Tartrate, Ammonium Nitrate and Oxalate, Calcium Phosphate, Magnesium Sulphate, Carbonate and Oxide, Abuminium Hydrate, Ferrous Sulphate, Ferric Sulphate and Hydrate, Copper Sulphate and

Lead Acetate. Part Second is devoted to Qualitative Analysis, and Part Third to Quantitative Analysis, together with a description of apparatus, and the processes of filtration, evaporation, crystallization, ignition, etc. The work extends over nearly one hundred pages, and is a valuable text-book for the student.

